# Controlling Nutritional Status (CONUT) Score as Prognostic Indicator in Stage IV Gastric Cancer with Chronic Intestinal Failure

**DOI:** 10.3390/nu16234052

**Published:** 2024-11-26

**Authors:** Konrad Matysiak, Aleksandra Hojdis, Magdalena Szewczuk

**Affiliations:** 1 Centre for Intestinal Failure, Poznan University of Medical Sciences, 60-355 Poznań, Poland; 2 Department of Gastroenterology, Poznan University Hospital, 60-355 Poznań, Poland; aleksandra.hojdis@gmail.com (A.H.); magdaszewczuk@wp.pl (M.S.)

**Keywords:** gastric cancer, chronic intestinal failure, CONUT, home parenteral nutrition

## Abstract

The management of chronic intestinal failure (CIF) secondary to advanced gastric cancer poses clinical challenges. This study explores the correlation between the Controlling Nutritional Status (CONUT) index and survival in patients with TNM stage IV gastric cancer on home parenteral nutrition (HPN). Methods: From 2015 to 2023, 410 patients (37% women, 63% men) with CIF due to advanced gastric cancer were assessed using CONUT scores, BMI, and biochemical tests. The Cox proportional hazards model was used to evaluate the impact of covariates on survival. Logistic regression categorized malnutrition levels by CONUT scores, with performance evaluated using precision, recall, and F1 scores. A *p*-value < 0.001 was statistically significant. Results: The CONUT scores were independent predictors of survival, with higher CONUT scores increasing mortality risk (HR = 2.073, 95% CI: 1.815–2.369, *p* < 0.001). The model achieved an overall accuracy of 71%, indicating correct classification for the majority of cases. Conclusions: CONUT scores are key predictors of survival in patients receiving HPN for CIF due to stage IV gastric cancer.

## 1. Introduction

Patients with cancer commonly experience malnutrition, which arises from multiple factors. It can result from inadequate food intake, malabsorption, and the prevalence of catabolic and pro-inflammatory processes. Additional contributing factors include extensive surgical procedures, chemotherapy toxicity, insufficient psychological support, and a declining quality of life. As cancer progresses, worsening malnutrition often leads to cachexia, a syndrome that negatively impacts patient survival outcomes [1,2].

Gastric cancer is the fifth most common malignancy and the fourth leading cause of cancer-related deaths worldwide [3]. The risk of developing malnutrition increases in advanced gastric cancer. This is due to disease progression and the presence of metastases in the abdominal cavity. These factors lead to impaired intestinal transit, malabsorption, and a reluctant or restricted food intake [2]. Understanding these factors is crucial for the development of precise tools to assess nutritional status, which in turn can lead to improved treatment outcomes for patients with advanced gastric cancer [4].

The Controlling Nutritional Status (CONUT) scale is an automatic, objective tool based on the analysis of biochemical tests and the total lymphocyte count. By measuring serum albumin levels, cholesterol, and the total lymphocyte count, the CONUT scale combines the assessment of the body’s protein reserves, caloric depletion, and immune defense [5]. The CONUT score, which employs commonly available laboratory methods to evaluate nutritional status, presents several advantages, such as the ease of obtaining results, low cost, reliability, and objectivity. Nevertheless, laboratory outcomes may be affected by factors unrelated to nutritional status, including systemic inflammatory response, liver insufficiency, surgical trauma, chemotherapy, and the type of tumor [6]. Previous studies on patients with gastric cancer have shown that a high preoperative CONUT score is linked to an increased risk of postoperative complications [7,8]. Additionally, patients with a high preoperative CONUT score have been found to have shorter overall survival (OS) and disease-free survival (DFS) following gastrectomy [9]. A high preoperative CONUT score indicating malnutrition has also been identified as an independent prognostic factor for OS in patients with stage II and III gastric cancer undergoing curative gastrectomy followed by adjuvant chemotherapy [10]. This study was conducted in a group of patients receiving home parenteral nutrition (HPN) due to chronic intestinal failure (CIF) [11] resulting from stage IV advanced gastric cancer, according to the TNM classification. The group consisted of patients receiving HPN at a single nutritional care center.

The aim of our study was to evaluate the effectiveness of the CONUT nutritional status index in predicting overall survival in patients with chronic intestinal failure due to stage IV advanced gastric cancer, according to the TNM classification, requiring HPN. 

## 2. Materials and Methods

### 2.1. Study Population

**Figure 1:**
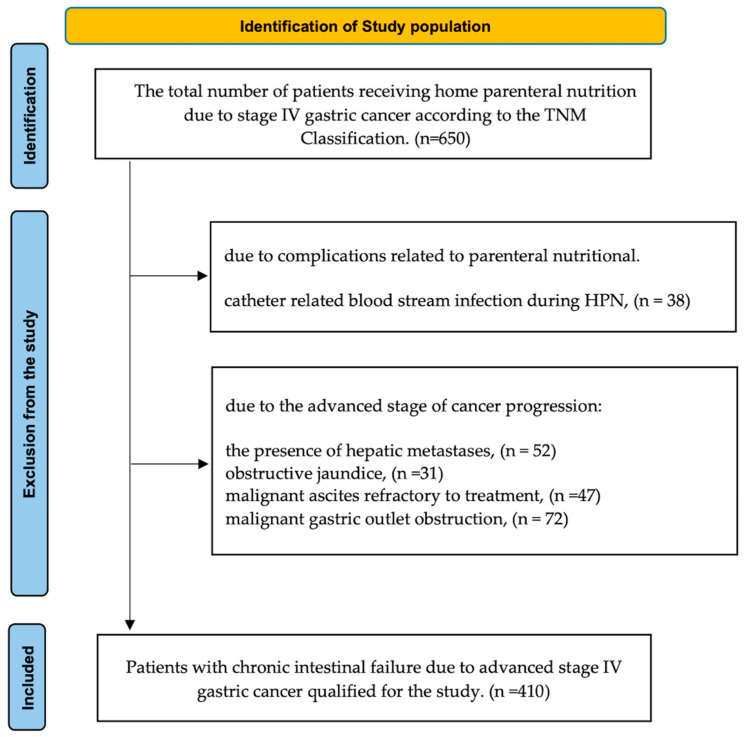
The flowchart diagram illustrates the total number of patients treated for chronic intestinal failure caused by advanced stage IV gastric cancer [12].

This retrospective study included patients whose HPN was initiated between 1 January 2015 and 31 December 2023. The observation period concluded on 31 July 2024. Only patients with advanced gastric cancer at TNM stage IV were eligible for analysis (Figure 1). The inclusion criteria were as follows:

- Advanced stage IV gastric cancer;
- Chronic intestinal failure preventing natural oral intake;
- The administration of HPN.

Exclusion criteria included the following:

- The presence of hepatic metastases;
- Obstructive jaundice;
- Malignant ascites refractory to treatment;
- Catheter-related blood stream infection during HPN;
- Malignant gastric outlet obstruction (MGOO);
- Lack of patient consent.

The hypothesis of this study posited that the CONUT nutritional index, assessed at the initiation of treatment, correlates with the overall survival of patients with stage IV gastric cancer. 

This study was conducted on a cohort of patients who developed chronic and irreversible intestinal failure as a consequence of stage IV advanced gastric cancer, with subsequent tumor infiltration involving the peritoneum, mesentery, and small intestine wall.

Intestinal failure is defined as a reduction in gut function to a level below the minimum required for the absorption of macronutrients—such as amino acids, fats, and carbohydrates—and/or water and electrolytes, necessitating intravenous supplementation to sustain health and/or promote growth.

Patients enrolled in this study met the following criteria for home parenteral nutrition:

- Inability to fulfil nutritional needs through oral and/or enteral routes;
- Confirmed metabolic stability;
- Health condition suitable for safe home-based management.

All patients enrolled in this study required parenteral nutrition due to chronic and irreversible intestinal failure. Nevertheless, they retained a partial ability to consume food orally, which, while insufficient to meet their full nutritional requirements, did satisfy their desire to eat. These patients received dietary guidance specifically tailored to their individual needs and capacities.

### 2.2. Data Collection

The patients’ nutritional status was assessed using the following:

- The CONUT score [5], which was derived from the following:
  a. Serum albumin (g/L);
  b. Total cholesterol (mg/dL);
  c. Total lymphocyte count (TLC).

TLC was calculated through a leukogram using the lymphocyte percentage and absolute lymphocyte count (mL).
According to THE CONUT nutritional index, patients were classified as follows:
  a. Those scoring 0 and 1 points were classified as well-nourished;
  b. Those scoring from 2 to 4 points were classified as mildly malnourished;
  c. Those scoring from 5 to 8 points were classified as moderately malnourished;
  d. Those scoring from 9 to 12 points were classified as severely malnourished.

- BMI, which was calculated using Quetelet’s formula.
- Biochemical assessment, which was performed in a certified university hospital laboratory according to standardized procedures and good laboratory practice. The evaluation included the following:
  a. Complete blood count;
  b. Hemoglobin (g/dL);
  c. White blood cells (×10^9^/L);
  d. Lymphocyte cells (×10^9^/L);
  e. Percentage of lymphocytes (%);
  f. Total protein (g/dL);
  g. Albumin (g/L);
  h. Total cholesterol (mg/dL).

The analysis included test results obtained on the day of hospital admission for qualification for home parenteral nutrition therapy.

### 2.3. Statistical Analyses

The median was employed as the central measure of variables to reduce the impact of outliers and provide a representative view of the data. This was further complemented by the interquartile range IQR. To analyze the differences between the four CONUT nutritional index classes, the Kruskal–Wallis statistical test was applied. The Cox proportional hazards regression model was used to evaluate the influence of patients’ age, gender, BMI, CONUT nutritional index on overall survival time. Key statistical metrics, including the confidence intervals (CI), hazard ratio (HR), and *p*-value, were considered to determine the significance of each covariate. To complement the Cox regression analysis, a logistic regression model was used to identify significant predictors of the four CONUT nutritional index classes. In the conducted logistic regression analysis, the model aimed to predict the assignment of cases to malnutrition degrees derived from the CONUT score. The model’s performance was evaluated in terms of precision, recall, and F1 score for each degree individually. An overall performance measure was also conducted through the evaluation of macro average and weighted average.

Kaplan–Meier survival curves were created to visualize survival probabilities for the four CONUT classes. The log-rank test was used to compare survival distributions between CONUT classes. The test statistic for the log-rank test, derived from a chi-squared distribution, was used to evaluate the statistical significance of differences between groups. A *p*-value < 0.001 was considered statistically significant. Statistical analysis was completed using Python 3.12.0 and the Lifelines package, while survival curves were generated using the Matplotlib package.

## 3. Results

### 3.1. Patient Characteristic

This study included 410 patients (153 women and 257 men) aged 28–93 years. The median (IQR) overall survival of the patients was 195 (285) days, ranging from 14 to 1262 days. All patients were confirmed to have stage IV adenocarcinoma of the stomach according to the TNM classification. The pathophysiological mechanism of chronic intestinal failure was classified as malignant mechanical obstruction in all cases. The mean volume and total calories administered during the study period per 24 h were 1721 ± 532 mL and 28.6 ± 6.5 kcal/kg. In 74 (18%) patients, gastrectomy was performed prior to the initiation of parenteral nutrition. In the study group, 170 (40%) patients received palliative 5-fluorouracil-based adjuvant chemotherapy concurrently with parenteral nutrition. The characteristics of the patients in relation to the applied treatment method are presented in Table 1. Selected parameters of the nutritional status assessment of the patients are detailed in Table 2.

### 3.2. Cox Regression Results

The Cox regression analysis revealed that among the covariates, the CONUT score was a statistically significant predictor of the hazard for the analyzed event. The hazard ratio (HR) for CONUT was 2.073 (95% CI 1.815–2.369; *p* < 0.001), indicating that higher CONUT levels more than doubled the hazard. Other covariates, including age, BMI, and sex, did not show statistically significant effects on the hazard, with their 95% confidence intervals encompassing the null value (HR = 1). Detailed results for these covariates are presented in Table 3.

### 3.3. Logistic Regression Results

The logistic regression analysis for the different malnutrition classes derived from the CONUT score yielded the following results. Well-nourished (n = 24): the model demonstrated a precision of 0.81 and a recall of 0.71, indicating that 71% of actual cases in this class were correctly identified. The F1 score of 0.76 suggests a balanced relationship between precision and recall within this class. Mild malnutrition (n = 32): the model achieved a precision of 0.71 and a recall of 0.75. The F1 score of 0.73 indicates that the model performed moderately well in predicting membership within this group. Moderate malnutrition (n = 22): a precision of 0.68 was observed, with a recall of 0.77, suggesting that the model exhibited good sensitivity in detecting cases within this class. The F1 score of 0.72 reflects reasonable overall performance for this category. Severe malnutrition (n = 4): the model’s performance for this class was notably poor, with precision, recall, and F1 score all at 0.00, indicating its inability to effectively classify cases in this group. Accuracy: the model achieved an overall accuracy of 71%, indicating that approximately 71% of all cases were correctly classified. Macro average: precision: 0.55; recall: 0.56; F1 score: 0.55. These results suggest that the model’s performance was uneven across the different classes, with a notable disparity in its ability to handle the smaller classes. Weighted average: precision: 0.69; recall: 0.71; F1 score: 0.70. These scores reflect better overall performance for the larger, more dominant classes within the dataset. 

**Figure 2:**
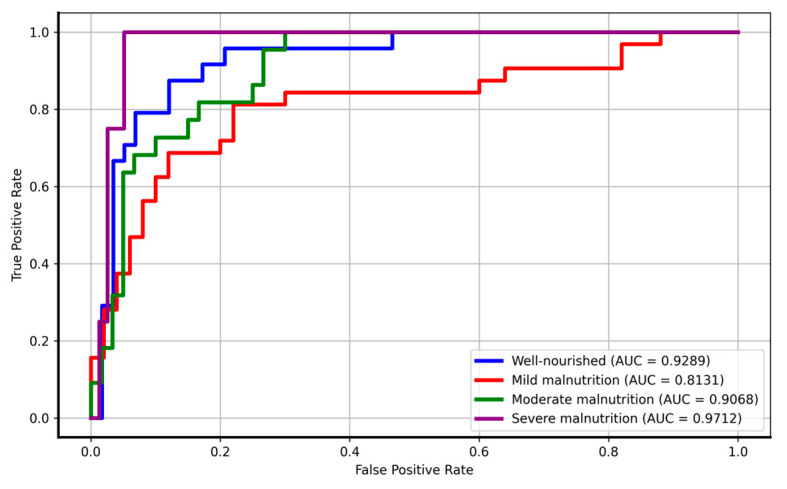
ROC curves for the logistic regression model across the four nutritional status categories.

The ROC curve (Figure 2) and the overall AUC score of 0.9050 collectively demonstrate that the model exhibits strong performance in distinguishing between the four nutritional status categories. The individual AUC scores, ranging from 0.8131 to 0.9712, indicate the model’s high discriminatory capability, effectively balancing sensitivity and specificity across the dataset. The well-nourished category demonstrates strong model performance, with a steep ROC curve reflecting high sensitivity and specificity, indicating accurate classification with minimal false positives. The severe malnutrition category achieves near-perfect discrimination, as evidenced by its steep and almost vertical curve. This result highlights the model’s ability to effectively classify this category, though it may be influenced by a small sample size. The moderate malnutrition category also shows strong performance, with a curve approaching the ideal top-left corner, signifying effective classification of this group. In contrast, the mild malnutrition category exhibits a slightly weaker performance, with a ROC curve suggesting a greater overlap with other categories, leading to higher false positive rates. Nevertheless, it still demonstrates reasonable classification ability, contributing positively to the overall AUC score. These results confirm that the model is highly effective at distinguishing nutritional status categories, with particularly strong performance for the severe malnutrition and well-nourished classes.

### 3.4. Kaplan–Meier Survival Curves and Log-Rank Test

**Figure 3:**
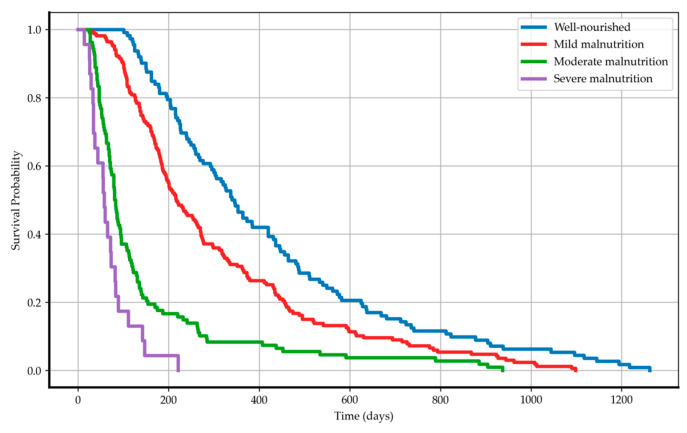
Kaplan–Meier curves for overall survival: stratified by CONUT classes.

To visualize the probability of survival for CONUT classes, Kaplan–Meier survival curves were generated (Figure 3). The Log-rank test results showed statistically significant differences: well-nourished versus mild malnutrition had a test statistic of 8.74 (*p*-value < 0.001; −log_2_(*p*) = 8.32); well-nourished versus moderate malnutrition had a test statistic of 82.87 (*p*-value < 0.001; −log_2_(*p*) = 63.31); and well-nourished versus severe malnutrition resulted in a test statistic of 169.79 (*p*-value < 0.001; −log_2_(*p*) = 126.52). The comparison between mild and moderate malnutrition produced a test statistic of 55.76 (*p*-value < 0.001; −log_2_(*p*) = 43.47), while mild versus severe malnutrition had a test statistic of 118.72 (*p*-value < 0.001; −log_2_(*p*) = 89.42). Lastly, moderate versus severe malnutrition showed a test statistic of 8.88 (*p*-value < 0.001; −log_2_(*p*) = 8.44). There are also statistically significant differences in survival between groups with serum albumin levels ≥ 3.5 g/dL and <3.5 g/dL and a test statistic of 62.74 (*p*-value < 0.001; −log_2_(*p*) = 48.59).

## 4. Discussion

The objective of this study was to evaluate the validity of the CONUT score as a predictor of overall survival in patients with chronic intestinal failure secondary to stage IV advanced gastric cancer, as classified by the TNM system.

In advanced gastric cancer, intestinal passage disorders are often due to tumor infiltration of the peritoneum, intestinal wall, and mesentery. These conditions are chronic and frequently lead to chronic intestinal failure. Additionally, these passage disorders are commonly associated with imbalances in water and electrolytes, third-space fluid retention, and persistent vomiting [11].

Malnutrition in cancer patients is a multi-etiological condition, contributing to the development of cachexia and significantly decreasing overall survival. In advanced gastric cancer, progressive cachexia frequently necessitates the discontinuation of surgical interventions, chemotherapy, and radiotherapy [1]. Consequently, cancer patients require continuous and automated monitoring of their nutritional status to ensure timely nutritional interventions and improve clinical outcomes [13]. Patients with advanced gastric cancer who are at risk of malnutrition, or are already affected by it, may benefit from early nutritional intervention to prevent perioperative complications and exclusion from adjuvant chemotherapy, and to avoid a reduction in overall survival [14].

Objective, automated prognostic parameters for survival in gastric cancer, which are based on immune system indicators such as the neutrophil-to-lymphocyte ratio (NLR) [15] and the lymphocyte-to-monocyte ratio (LMR) [16], have been extensively characterized. It has been demonstrated that the preoperative lymphocyte-to-monocyte ratio is an independent prognostic factor for survival in patients with resectable gastric cancer [16]. Furthermore, in an exploratory analysis conducted to assess the prognostic value of the neutrophil-to-lymphocyte ratio in patients with esophageal and gastric cancer, it was shown that a high NLR was associated with shorter overall survival and an increased risk of mortality [15]. The importance of both the neutrophil-to-lymphocyte ratio and the lymphocyte-to-monocyte ratio as components of the Naples Prognostic Score in predicting survival has also been demonstrated in patients with remnant gastric cancer undergoing surgery. [17]. In the studied patient cohort, it was observed that the median lymphocyte count and the total lymphocyte number decreased with the increasing degree of malnutrition, as assessed by the CONUT score. This may indicate a weakened immune response in malnutrition, an ineffective anti-tumor attack by lymphocytes, and an inability to directly destroy tumor cells [18]. This phenomenon is associated with poorer overall survival and disease-free survival outcomes [19]. 

In our study, we chose to employ the CONUT score, which, similarly to the Naples Prognostic Score [20], evaluates components of the immune system. Additionally, it incorporates biochemical serum parameters, such as albumin concentration and total cholesterol level, which are crucial for assessing patients’ nutritional status.

The CONUT score offers an automated, objective, and easily verifiable method for assessing patients’ nutritional status [5]. This evaluation helps identify patients who require frequent monitoring, nutritional support, or adjustments to their nutritional intervention. As demonstrated, the CONUT score is an effective tool for evaluating the nutritional status of patients with gastric cancer, and its predictive utility for survival outcomes has been well documented. Previous studies have shown that patients with higher CONUT scores in stages I, II, and III of gastric cancer had significantly shorter survival times [21,22]. In our study, the CONUT score assessed prior to the initiation of HPN was identified as an independent prognostic factor for overall survival in patients with stage IV gastric cancer, as classified by the TNM system.

Our study has several limitations. First, it was conducted in a single nutritional center, which, despite the large sample size, may have led to an uneven distribution across the different CONUT score classifications. Our model demonstrates very high accuracy for severe malnutrition and well-nourished individuals, high accuracy for moderate malnutrition, and moderate accuracy for mild malnutrition. In our opinion, the high predictive value for severe malnutrition observed in the studied cohort may be attributable to the limited number of patients with this degree of malnutrition included in this study. However, we believe that the predictive effectiveness for overall survival in patients with chronic intestinal failure could be further enhanced by expanding the study population and incorporating additional markers into the analysis. These could include tumor process markers such as CEA and CA 19-9, and novel biomarkers like microRNAs, including miR-106. Such an approach would provide a more comprehensive understanding and improve the predictive accuracy of the model [23].

Despite the CONUT score, based on objective biochemical test results and lymphocyte counts, demonstrating high reliability in both prospective and retrospective analyses, the retrospective nature of this study limited its evaluation of prognostic effectiveness in assessing overall survival when compared with widely used subjective screening tools, such as NRS 2002 [24], PG-SGA, and NUTRISCORE [25].

## 5. Conclusions

The findings of this analysis indicate that the CONUT score is a significant predictor of overall survival in patients receiving home parenteral nutrition (HPN) due to chronic intestinal failure resulting from advanced gastric cancer at stage IV, according to the TNM classification.

It was observed that an increase in the CONUT score is associated with an exponential rise in the risk of reduced overall survival in the studied cohort. Higher CONUT scores correlated with a progressively greater risk of the analysed event.

These results underscore the importance of regular nutritional status monitoring, which could help mitigate the adverse clinical impacts of malnutrition, improve prognostic outcomes, and extend overall survival in the studied population.

## Figures and Tables

**Table 1:** The table presents the characteristics of the patients in relation to the CONUT stage and the applied treatment method (n = 410).

Nutritional Status According to CONUT Score	Well Nourished (*n* = 112)	Malnutrition			*p*-Value
		Mild (*n* = 167)	Moderate (*n* = 108)	Severe (*n* = 23)	
Chemotherapy	26	45	23	2	0.240
Gastrectomy and chemotherapy	20	25	26	3	0.250
Parenteral nutrition only	66	97	59	18	0.223

The actual data represent the number of patients in the respective groups. CONUT: Controlling Nutritional Status.

**Table 2:** The table presents the distribution of selected parameters across the classes of the CONUT scale (n = 410).

Nutritional Status According to CONUT Score	Well Nourished (*n* = 112)	Malnutrition			*p*-Value
		Mild (*n* = 167)	Moderate (*n* = 108)	Severe (*n* = 23)	
White blood cells (×10^9^/L)	8 (4)	8 (5)	8 (7)	11 (7.5)	0.208
Lymphocyte cells (×10^9^/L)	2 (0.8)	1.5 (0.8)	1.1 (0.8)	1.1 (0.5)	<0.001
% Lymphocytes	26.6 (10.7)	17.8 (12.7)	11.9 (11.3)	7.7 (6.6)	<0.001
Cholesterol total (mg/dL)	184 (43.4)	148 (52.5)	134 (37)	110 (41)	<0.001
Albumin (g/dL)	4 (0.6)	3.7 (0.7)	3.1 (0.6)	2.4 (0.4)	<0.001
Protein total (g/dL)	7 (0.9)	6.8 (0.8)	6.2 (1)	5.4 (1)	<0.001
Hemoglobin (g/dL)	11.7 (1.9)	11.3 (2.1)	10.4 (1.9)	9.4 (0.9)	0.633
TLC cells/m^3^	1974 (806)	1400 (878)	960 (714)	889 (589)	<0.001
BMI kg/m^2^	20.2 (5.4)	19.9 (7.9)	19.1 (4.8)	21.3 (4.7)	0.283

Data are presented as median (interquartile rate). CONUT: Controlling Nutritional Status; TLC: total lymphocyte count; BMI: body mass index.

**Table 3:** Cox regression analysis of prognostic factors for overall survival.

Covariate	HR	95% CI	*p*-Value
		Lower–Upper	
Age (years)	0.992	0.983–1.001	0.084
Sex	1.112	0.898–1.376	0.329
BMI (kg/m^2^)	0.984	0.958–1.010	0.225
CONUT Stage	2.073	1.815–2.369	<0.001

HR: hazard ratio; CI: confidence interval; CONUT: Controlling Nutritional Status; BMI: body mass index.

## Data Availability

The data presented in this study are available on request from the corresponding author for any academic use upon citation of this article. The data are not publicly available due to privacy and permission constraints that restrict their use to the publication of this article only.
